# Alleviating the Hydrolysis of Carbohydrates, Tangzhiqing (TZQ) Decreased the Postprandial Glycemia in Healthy Volunteers: An Eight-Period Crossover Study

**DOI:** 10.1155/2020/8138195

**Published:** 2020-03-16

**Authors:** Yanfen Li, Ziqiang Li, Ruihua Wang, Bo Mi, Ting Jiang, Meijuan Lu, Jia Liu, Baohe Wang, Deqin Zhang, Qiang Xu, Yuhong Huang

**Affiliations:** ^1^Second Affiliated Hospital of Tianjin University of Traditional Chinese Medicine, No. 69 Zengchan Road, Hebei District, Tianjin 300250, China; ^2^Tianjin University of Traditional Chinese Medicine, No. 10 Poyanghu Road, Tuanbo New Town, Jinghai District, Tianjin 301617, China

## Abstract

Tangzhiqing (TZQ), a Chinese herbal medicine, has been widely used to treat diabetes mellitus in China. TZQ works as a potential *α*-glucosidase inhibitor to reduce the absorption of glucose from dietary carbohydrates. The main aim of this study was to investigate the postprandial glucose-lowering effect of TZQ on the common carbohydrates in healthy humans. Meanwhile, the possible types of the inhibited *α*-glucosidase enzymes were predicted in this study. Glucose, sucrose, maltose, maltodextrin, and starch were chosen as investigated carbohydrates. The baseline incremental area under the curve (IAUC) and glycemic index (GI) values of the investigated carbohydrates were evaluated. Then, thirty-six subjects were randomly assigned to three groups to assess postprandial hypoglycemic effects of 3-, 6-, and 9-tablet TZQ. The subjects in each group were randomized to eight subgroups. An eight-period, eight-sequence, crossover design was performed to investigate the postprandial glucose-lowering effect of TZQ after drinking each carbohydrate. A significant decrease was observed on the postprandial glucose IAUCs (279.41 ± 111.31 vs. 203.86 ± 61.08) and GIs (124.91 ± 48.54 vs. 91.69 ± 27.47) of maltose after oral administration of 6-tablet TZQ, as well as IAUCs (145.05 ± 55.01 vs. 110.23 ± 57.03) and GIs (84.87 ± 33.40 vs. 65.50 ± 33.89) of sucrose after administration of 3-tablet TZQ. The glucose IAUCs (109.15 ± 55.92 vs. 57.68 ± 46.09) and GIs (49.09 ± 25.15 vs. 25.94 ± 20.73) of starch statistically reduced following the administration of 6-tablet TZQ. The lowering postprandial blood glucose effect of TZQ did not increase proportionally with increasing doses in humans. There were no significant changes in the glucose-lowering effect of glucose and maltodextrin after the administration of 3-, 6-, or 9-tablet TZQ, respectively. TZQ is a potential treatment for postprandial hyperglycemia, which can probably make *α*-glucosidases inhibit maltase, sucrase, and *α*-amylase in the digestive organs.

## 1. Background

The number of people globally with diabetes mellitus is projected to rise to 439 million by 2030, which represents 7.7% of the total adult population of the world [1, 2]. The rapidly increasing diabetes mellitus is becoming a serious threat to human health all over the world [3]. The elevated postprandial blood glucose is one of the earliest abnormalities of glucose homeostasis associated with diabetes mellitus [4]. It was reported that traditional Chinese medicines possessed a significant postprandial hypoglycemic effect [5]. However, the effect and mechanism of TZQ were still not well known on the postprandial glycemia resulting from carbohydrates.

TZQ, a Chinese herbal medicine, has been broadly used to treat diabetes mellitus. It was composed of *Nelumbo nucifera* Gaertn. leaf, *Paeonia lactifiora* Pall. root, *Salvia miltiorrhiza* Bge. root, *Morus alba* L. leaf, and *Crataegus pinnatifida* Bge. leaf [6, 7]. In the genetically type 2 diabetes KK-A^y^ mice, TZQ presented beneficial effects on the improvement of the glucose metabolism by reducing the *α*-glycosidase activity [6]. The TZQ formula and its main fractions showed strong inhibitory effects on the small intestinal *α*-glucosidases of maltase and sucrase in rats [7, 8]. In healthy volunteers, 3-tablet TZQ had the same effect as the acarbose, an *α*-glucosidase inhibitor, to decrease the maximum concentrations of plasma glucose after breakfast and dinner [9]. The glucose-lowering effect appeared to increase proportionally with increasing TZQ dose based on the qualitative, quantitative, and dose-exposure-response analysis in humans [10, 11]. In addition, TZQ could reduce glycosylated hemoglobin (HbA1c) and fasting insulin in type 2 diabetes mellitus patients [12].

One of the therapeutic mechanisms on diabetes mellitus is to retard the absorption of glucose via the inhibition of *α*-glucosidases in the small intestine. The *α*-glucosidases, including maltase, sucrase, lactase, *α*-dextrinase, and *α*-amylase, distributed widely in the epithelium of the small intestine [13, 14]. Many national and international guidelines recommend the use of *α*-glucosidase inhibitors as a first-line alternative to metformin or in combination with sulfonylureas, metformin, other newer oral hypoglycemic agents, or insulin. The *α*-glucosidase inhibitors could depress the absorption of glucose by hindering the hydrolytic cleavage of disaccharides, oligosaccharides, and polysaccharides into absorbable monosaccharides in the digestive process of carbohydrates [15–18]. Since TZQ was a potential *α*-glucosidase inhibitor, it was possible that TZQ can retard the liberation of glucose from dietary complex carbohydrates and delay glucose absorption [6–8]. Nonetheless, the possible types of *α*-glucosidases inhibited by TZQ had not been investigated.

In this study, a randomized, eight-period, crossover design was performed to assess the plasma glucose profiles after the administration of glucose, maltose, sucrose, maltodextrin, or starch with and without TZQ, respectively. The postprandial lowering-glucose effects of 3-, 6-, and 9-tablet TZQ were evaluated by the plasma glucose glycemic indexes (GIs) and incremental area under the curves (IAUC) in healthy volunteers who drank one of five investigated carbohydrates. Moreover, the types of *α*-glucosidase enzymes inhibited by TZQ were predicted according to the changes of GIs.

## 2. Materials and Methods

### 2.1. Materials

Tangzhiqing tablets (TZQ, 0.64 g) were produced by Buchang Shenzhou Pharmaceutical Co., Ltd. (Shandong, China). The contents of paeoniflorin and nuciferine in the TZQ (Lot number: 160301) were 15.1 mg and 1.34 mg, respectively. Glucose, sucrose, maltose, maltodextrin, and starch were obtained from Xiwang Pharmaceutical Co., Ltd. (Shandong, China), TS Corporation (Seoul, Korea), Tianjiu Biotechnology Co., Ltd. (Shandong, China), Xiwang Sugar Co., Ltd. (Shandong, China), and Wuhu Haoyikuai Food Co., Ltd. (Anhui, China), respectively.

### 2.2. Study Participants

The study population comprised healthy Chinese male and female volunteers aged 18 to 40 years with a body mass index between 19 and 25 kg/m^2^. Screening included an evaluation of laboratory analysis of hepatic and renal function, hematologic profile, and disease markers for HIV and hepatitis B and C viruses, medical history, substance use history, chest radiography, 12-lead ECG, physical examination, and demographic data. The fasting glucose level was 3.9–5.5 mmol/L (70–100 mg/dL), and the glycosylated hemoglobin value was less than 6%. Only medically healthy subjects with clinically normal laboratory profiles were enrolled. Exclusion criteria were signs or symptoms of organ dysfunction, allergy, or intolerance to any study medication, a known history of diabetes mellitus or the use of antihyperglycemic drugs or insulin to treat diabetes and related conditions, and the use of CYP450s modifying drugs within 30 days.

### 2.3. Study Design

According to the Joint FAO/WHO Expert Consultation [19], six more subjects were needed to assess the blood glucose response of carbohydrates in human nutrition. A minimum of 10 healthy subjects was required for the determination of the glycemic index and recommendation for food classification in accordance with the provision of ISO 26642 : 2010 (E) standard [20]. For this trial, 12 healthy subjects were enrolled in each group to evaluate the acute effects of TZQ on the postprandial glycemia of five carbohydrates.

Firstly, an open-label, four-period, paralleled study design was implemented to assess the baseline glycemic indexes of sucrose, maltose, maltodextrin, and starch. Afterwards, thirty-six subjects were randomly assigned to three groups and each group enrolled 12 participants according to a randomized block design. The influences of 3-, 6-, and 9-tablet TZQ on the blood glucose levels were evaluated after the oral administration of carbohydrates to subjects in the *low-dose group*, *middle-dose group*, and *high-dose group*, respectively. Then, the subjects in each group were randomly assigned to eight subgroups in accordance with a randomized block design. Each subgroup enrolled 2 participants in four subgroups, whereas each subgroup enrolled one participant in other subgroups (Table 1). In each subgroup, a randomized, eight-cycle, eight-sequence, crossover design was performed to investigate the effects of TZQ on the glycemic indexes of glucose, sucrose, maltose, maltodextrin, and starch. Among them, glucose was given three times as a reference food according to the Joint FAO/WHO Expert Consultation [19].

The protocol and informed consent form were approved by the Ethics Committees of the Second Affiliated Hospital of Tianjin University of Traditional Chinese Medicine (ethical approval no. 2016-007-01). The clinical trial was conducted at the Clinical Practice Center of the Second Affiliated Hospital of Tianjin University of Traditional Chinese Medicine (Tianjin, China). This study was performed in accordance with the principles of the Declaration of Helsinki and the Guidelines of CONSORT.

### 2.4. Carbohydrates and Drug Administration

All participants provided informed consent by signing a written consent form before the screening. Subjects were admitted to the clinical trial center on the evening before the day of drug and/or carbohydrate administration. The participants were asked to fast for at least 10 hours overnight and avoid consumption of phytochemical-rich foods until the completion of the study. Before each dosing, 50 g of carbohydrate was freshly dissolved with 150 mL of water.

In the baseline assessment, subjects were consecutively given 50 g of maltose, sucrose, maltodextrin, and starch to assess the baseline glycemic indexes at the visit time of days 1, 4, 7, and 10, respectively. There was a 3-day washout between every two periods. Afterwards, subjects treated with 3-, 6-, and 9-tablet TZQ were arranged to *low-dose group*, *middle-dose group*, and *high-dose group*, respectively. Then, the subjects in each group were randomly assigned to eight subgroups. The dosing sequences of the eight interventions at the eight subgroups were presented in Table 1 with a 5-day washout period. TZQ tablets were immediately administrated after the consumption of 50 g of sucrose, glucose, maltose, maltodextrin, or starch in 5 minutes. In each dosing sequence, 50 g of glucose was individually given three times as a reference food in accordance with the joint FAO/WHO Expert Consultation *Carbohydrates in human nutrition* [19] and the International Organization for Standardization *Determination of the glycaemic index (GI) and recommendation for food classification* [20].

After the collection of blood samples at -15 min (time zero), an assigned drink was consumed within a 5-minute timeframe. At each study visit, blood samples were gathered before the drug administration and at 15, 30, 45, 60, 90, and 120 min after consuming carbohydrate foods to assess the glycemic indexes. Blood glucose concentrations were measured in capillary blood (finger prick) by a blood glucose meter (Johnson & Johnson, New Jersey, USA) with the dry glucose oxidase method. The type of blood sampling should be consistent within any one series of testing.

### 2.5. Data Analysis

The incremental glycemia at the time points after dosing different carbohydrates was calculated by subtraction of the fasting value (the blood glucose at time 0) [20]. The blood glucose response profiles were plotted as the change in blood glucose values from the fasting value on the ordinate. The average blood glucose response curves were plotted by calculating the mean blood glucose concentrations of all subjects at each time point. The graphs were plotted showing the glucose responses of the five carbohydrates with and without TZQ. The blood glucose response to the reference food was expressed as the incremental area under the curve (IAUC).

For an individual subject, the GI of the test carbohydrate (GI_t_) was expressed as follows [19, 20]:

(1) $$\begin{array}{c} {\text{GI}}_{t}=\frac{{\text{IAUC}}_{t}}{\bar{{\text{IAUC}}_{\text{ref}}}}\times 100, \end{array}$$

where IAUC_t_ was the IAUC of the test carbohydrate and IAUC_ref_ was the mean IAUC of the reference carbohydrate (glucose). The IAUC was the area under the curve calculated as the incremental area under the blood glucose response curve by applying the trapezoid rule and ignoring the area beneath the fasting concentration.

### 2.6. Statistical Analysis

All the results were presented as mean ± standard deviation. Descriptive statistics including arithmetic mean, standard deviation, and the number of observations were calculated for the vital sign measurements and blood glucose responses. Differences among the three groups were analyzed by one-way analysis of variance (ANOVA) for demographic indicators. Counting data was checked by chi-square. Differences in the parameters of IAUCs and GIs between the carbohydrate alone groups and the TZQ-treated groups were examined using paired *t*-test. For all analyses, the effects were considered statistically significant if their probability was *P* < 0.05. All statistical analyses were performed using SAS version 9.4 (SAS Institute Inc., North Carolina, USA).

## 3. Results

### 3.1. Study Population

**Figure 1:**
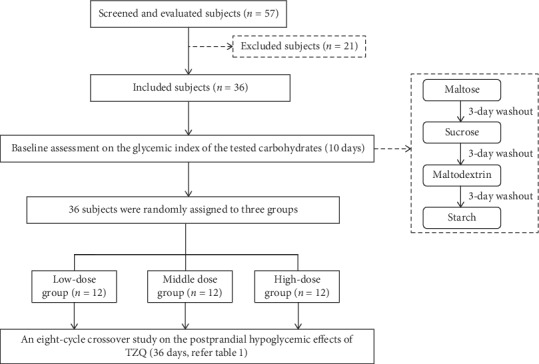
Flow diagram of the clinical trial.

A total of 57 subjects were screened for inclusion; 21 subjects were excluded prior to study initiation (Figure 1). Thirty-six healthy Chinese males and females were randomly assigned to the *low-dose group* (age, 25.83 ± 1.34 years; BMI, 21.89 ± 1.62 kg/m^2^), *middle-dose group* (age, 26.00 ± 2.45 years; BMI, 21.11 ± 1.58 kg/m^2^), and *high-dose group* (age, 25.50 ± 1.57 years; BMI, 21.53 ± 1.77 kg/m^2^). The baseline demographic and physiological characteristics of the healthy subjects in each group were summarized in Table 2. There were no statistically significant differences among the three groups on age, BMI, body temperature, pulse, breathing, systolic pressure, and diastolic pressure (*P* > 0.05). All of the subjects completed the study. The statistical analysis was performed by the original assigned groups.

### 3.2. Effect of TZQ on the Glycemic Index of Glucose

**Figure 2:**
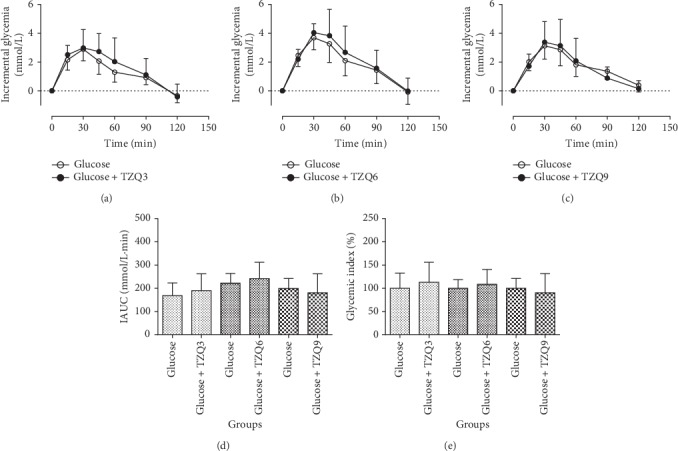
Average profiles of blood glucose response in humans after drinking glucose solution (50 g glucose/150 mL water) with and without the intervention of 3, 6, or 9-tablet TZQ.

Figure 2 shows the postprandial hypoglycemic effects of TZQ after drinking glucose solution (50 g glucose/150 mL water) with or without TZQ in healthy humans. The glucose-lowering effects of 3-, 6-, and 9-tablet TZQ on the blood glucose response are displayed in Figures 2(a), 2(b), and 2(c), respectively. Compared with the group of glucose alone, there was no significant difference in the incremental blood glucose profiles after the treatment with different doses of TZQ (*P* > 0.05). No statistic differences were observed on the IAUC values (168.26 ± 54.70 vs. 190.12 ± 72.74, 222.35 ± 41.80 vs. 241.76 ± 70.65, and 199.56 ± 42.62 vs. 180.46 ± 82.08) after its combination with 3-, 6-, and 9-tablet TZQ, respectively (*P* > 0.05, Figure 2(d)). The glycemic index values presented consistent results as the IAUC between the glucose group and the TZQ-treated group (*P* > 0.05, Figure 2(e)), suggesting that TZQ had no significant postprandial hypoglycemic effect after the oral administration of glucose.

### 3.3. Effect of TZQ on the Glycemic Index of Maltose

**Figure 3:**
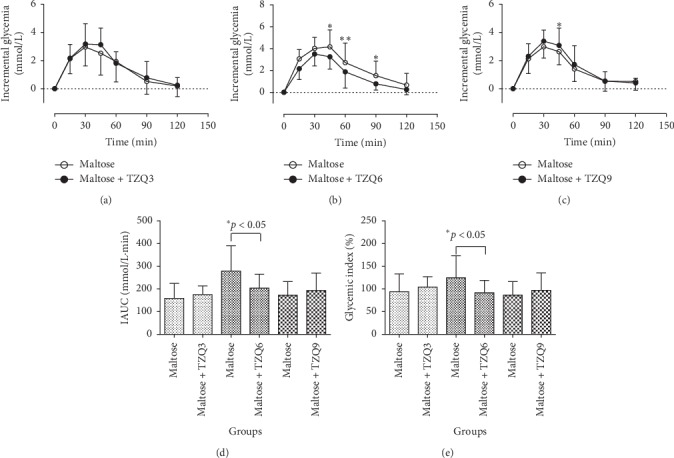
Average profiles of blood glucose response in humans after drinking maltose solution (50 g maltose/150 mL water) with and without the intervention of 3, 6, or 9-tablet TZQ.

The average profiles of postprandial glycemic responses in human are presented in Figures 3(a), 3(b), and 3(c) after drinking maltose solution (50 g maltose/150 mL water) with or without the intervention of 3-, 6-, and 9-tablet TZQ, respectively. Concurrent administration of 6-tablet TZQ reduced the incremental blood glucose at 45 min (*P*=0.042), 60 min (*P*=0.007), and 90 min (*P*=0.039). Besides, a significant decrease was observed on the blood glucose response at 45 min after the combination with 9-tablet TZQ (*P* < 0.01). Compared with the group of maltose alone, there were significant declines on the parameters of IAUCs (279.41 ± 111.31 vs. 203.86 ± 61.08, *P* < 0.05, Figure 3(d)) and glycemic indexes (124.91 ± 48.54 vs. 91.69 ± 27.47, *P* < 0.05, Figure 3(e)) following the treatment with 6-tablet TZQ. However, no statistical differences were observed on the parameters of IAUCs and glycemic indexes after the treatment with 3- or 9-tablet TZQ. The results indicated that TZQ had a potential inhibitory effect on the intestinal maltase at an appropriate dosage.

### 3.4. Effect of TZQ on the Glycemic Index of Sucrose

**Figure 4:**
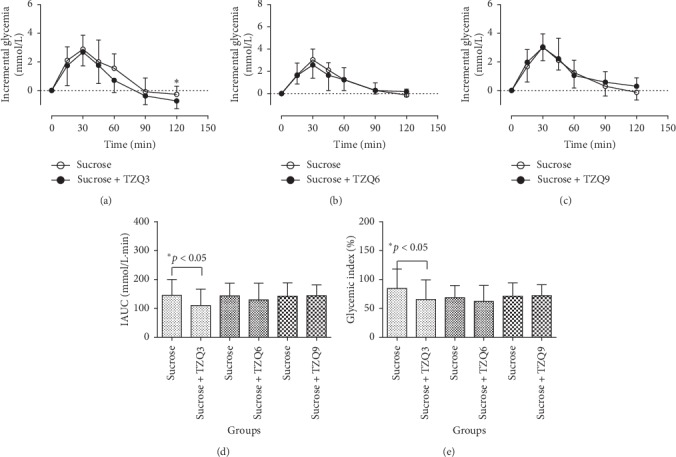
Average profiles of blood glucose response in humans after drinking sucrose solution (50 g sucrose/150 mL water) with and without the intervention of 3, 6, or 9-tablet TZQ.

The mean postprandial glycemic response profiles are presented in Figures 4(a), 4(b), and 4(c) after drinking 50 g sucrose solution (50 g sucrose/150 mL water) with or without the interventions of 3-, 6-, and 9-tablet TZQ, respectively. Compared with the group of sucrose alone, statistical reductions were observed on the incremental glycemia at 120 min after the concurrent administration of 3-tablet (*P*=0.026) or 9-tablet TZQ (*P*=0.021) with sucrose. Moreover, significant reductions were observed on the parameters of IAUCs (145.05 ± 55.01 vs. 110.23 ± 57.03, *P* < 0.05, Figure 4(d)) and glycemic indexes (84.87 ± 33.40 vs. 65.50 ± 33.89, *P* < 0.05, Figure 4(e)) following the treatment with 3-tablet TZQ. However, there were no significant differences in the parameters of IAUCs and glycemic indexes after the concurrent administration of 6- or 9-tablet TZQ. The results suggested that 3-tablet TZQ presented a more significant inhibitory effect than 6- or 9-tablet TZQ on the postprandial hyperglycemia.

### 3.5. Effect of TZQ on the Glycemic Index of Maltodextrin

**Figure 5:**
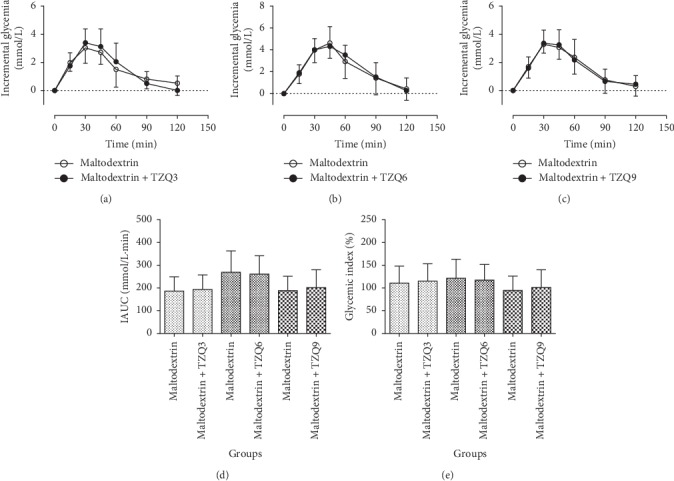
Average profiles of blood glucose response in humans after drinking maltodextrin solution (50 g maltodextrin/150 mL water) with and without the intervention of 3, 6, or 9-tablet TZQ.

Figures 5(a), 5(b), and 5(c) display changes of blood glucose responses in humans after the drinking of maltodextrin solution (50 g maltodextrin/150 mL water) with or without the interventions of 3-, 6-, and 9-tablet TZQ, respectively. No significant differences were observed on the incremental glycemia profiles with and without the intervention of TZQ (*P* > 0.05). No statistic differences were observed on the IAUC values (186.13 ± 63.41 vs. 193.29 ± 64.35, 269.17 ± 93.58 vs. 260.83 ± 80.73, and 188.59 ± 63.04 vs. 202.00 ± 77.75) after its treatment with 3-, 6-, and 9-tablet TZQ, respectively (*P* > 0.05, Figure 5(d)), as well as the glycemic index values (*P* > 0.05, Figure 5(e)).

It suggested that TZQ had no significant postprandial hypoglycemic effect in humans after the oral administration of maltodextrin.

### 3.6. Effect of TZQ on the Glycemic Index of Starch

**Figure 6:**
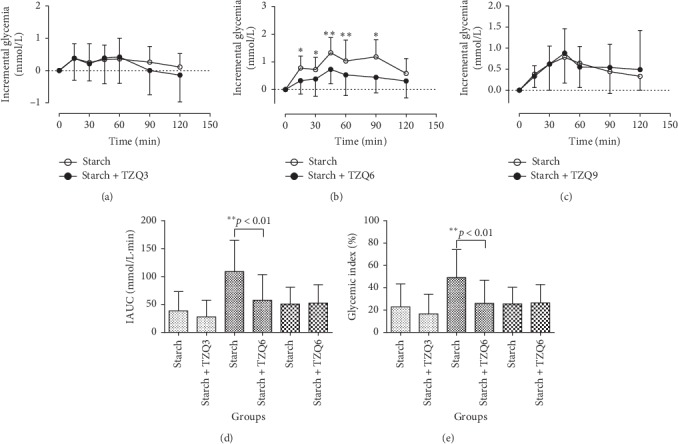
Average profiles of blood glucose response in humans after drinking starch solution (50 g starch/150 mL water) with and without the intervention of 3, 6, or 9-tablet TZQ.

The mean lowering-glucose effects of 3-, 6-, and 9-tablet TZQ on the incremental glycemia of starch solution (50 g starch/150 mL water) in humans are revealed in Figures 6(a), 6(b), and 6(c), respectively. After its treatment with 6-tablet TZQ, significant reductions were measured on the changes of postprandial blood glucose responses of starch at the time of 15 min (*P*=0.014), 30 min (*P*=0.04), 45 min (*P*=0.003), 60 min (*P*=0.007), and 90 min (*P*=0.02). Therefore, statistical decreases were observed on the parameters of IAUCs (109.15 ± 55.92 vs. 57.68 ± 46.09, *P*=0.01, Figure 6(d)) and glycemic indexes (49.09 ± 25.15 vs. 25.94 ± 20.73, *P*=0.05, Figure 6(e)) following the treatment with 6-tablet TZQ. However, there were no significant differences in the postprandial hypoglycemic effects after the concurrent administration of 3- or 9-tablet TZQ.

### 3.7. Tolerability

No serious adverse events were observed throughout the study. There were two transient and mild adverse events, stuffy nose and nausea. None of the adverse events were considered to be associated with the administration of carbohydrates or TZQ. None of the subjects withdrew from the study due to the observed adverse events. All other clinical evaluations, such as physical examinations, vital signs, ECGs, and clinical laboratory tests, were within the normal range.

## 4. Discussions

TZQ was composed of eight fractions, including mulberry leaf polysaccharide, lotus leaf flavonoids, mulberry leaf flavonoids, hawthorn leaf flavonoids, lotus leaf alkaloids, mulberry leaf alkaloids, red peony saponins, and Danshen polyphenols [7]. Many phytoconstituents, such as flavonoids, alkaloids, terpenoids, anthocyanins, glycosides, and phenolic compounds, possessed the *α*-glucosidase inhibitory potency [21, 22]. In our previous study, the fingerprint analysis of TZQ was carried out through UPLC-Q-TOF/MS assay [10]. Multicomponent quantitative analysis was implemented to the main ingredients of nuciferine, paeoniflorin, salvianolic acid B, hyperoside, and rutin by LC analysis [10]. Plasma paeoniflorin and nuciferine exhibited the appropriate pharmacokinetic properties in humans, including dose-dependent systemic exposure, and a proper elimination half-life (1∼3 h) [11]. The chemical markers of hyperoside, salvianolic acid B, and rutin could hardly be detected in human plasma after oral administration of 2.56-gram TZQ under the LC-MS/MS condition [11]. In addition, the short half-life characteristics (less than 8 h) of some other ingredients have been reported in literatures [23–26]. Therefore, a 5-day washout time between two periods was designed in this crossover study. Some *in vitro* studies revealed that TZQ displayed a powerful inhibitory action on the intestinal *α*-glucosidases [6], as well as the fractions of hawthorn leaf flavonoids [7], mulberry leaf alkaloids [27, 28], mulberry leaf flavonoids [29], lotus leaf flavonoids [30], and so on. Additionally, TZQ-treated KK-Ay mice showed a reduction in the sucrase and maltase activity (47%∼45% and 88%∼51%, respectively) after a 4-week treatment with a dose range from 0.175 to 0.7 g/kg. But TZQ did not reduce the lactase activity in the small intestine of KK-Ay mice [6]. The IC_50_ values of mulberry leaf alkaloids were 0.05 g/ml for maltase and 0.26 g/ml for sucrase, less than the positive control acarbose (0.75 and 0.69 g/ml, respectively) [7]. Particularly, 1-deoxynojirimycin (DNJ), which was abundant in mulberry leaf, was believed to be a typical naturally occurring iminosugar with potent biological activity similar to acarbose [28]. The mulberry leaf flavonoids showed *α*-glucosidase inhibitory activities, with the active constituents of rutin and astragalin showing high *α*-glucosidase inhibitory activities (IC_50_ values of 13.19 ± 1.10 and 15.82 ± 1.11 *μ*M, respectively) [29]. In addition, Yuhong et al. reported that 3 tablets of TZQ have the same effects as the acarbose [9]. Therefore, we made an assumption that TZQ was an effective therapeutic manner to reduce the postprandial plasma glucose as the acarbose, a typical *α*-glucosidase inhibitory.

The main objective of this study was to elucidate the hypoglycemic effects and the possible mechanisms of TZQ on postprandial glucose after drinking the common carbohydrates in Chinese healthy subjects. It was reported that TZQ possessed a potential regulation effect on abnormal glucose metabolism [6–8]. Yuhong et al. had compared the effect of lowering blood glucose between TZQ and acarbose in healthy humans [9]. The results presented that 50 mg of acarbose as well as 3 and 4 tablets of TZQ decreased the *C*_max_ of plasma glucose after dinner. Compared with the ingestion of maltose alone, a significant decrease was presented on the plasma glucose IAUC (279.41 ± 111.31 vs. 203.86 ± 61.08) and GI (124.91 ± 48.54 vs. 91.69 ± 27.47) after the concurrent administration of maltose with 6-tablet TZQ in this study (*P* < 0.05, Figure 3). Similarly, statistical reductions were observed on the glucose IAUC and GI values of sucrose after taking 3-tablet TZQ (*P* < 0.05, Figure 4), and after the concurrent ingestion of starch with 6-tablet TZQ (*P* < 0.01, Figure 6). The hypoglycemic effect of 6-tablet TZQ was superior to 3-tablet TZQ in healthy volunteers, which was consistent with the previous report that the glucose-lowering effect of TZQ appeared to increase proportionally within the measured doses ranging from 2 to 4 tablets [10, 11]. However, the hypoglycemic effect of 9-tablet TZQ was weaker than 6-tablet TZQ after the ingestion of maltose, sucrose, and starch. The reduced pharmacological activity of TZQ might result from the ceiling effect [31], or the antagonistic actions among the various components contained in the herbal medicine [32]. A further study will be designed to explore the specific reasons behind the phenomenon.

**Figure 7:**
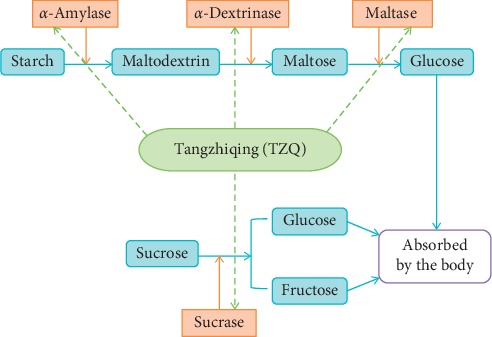
Schematic diagram of hydrolysis by glycosidase promotion and inhibition.

The *α*-glucosidase inhibitors were effective in almost all patients with high postprandial hyperglycemia as monotherapy or in combination with other oral hypoglycemic agents or insulin [33, 34]. The *α*-glucosidase enzymes could hydrolyze the glycosidic bonds of different carbohydrates by internal or external breakage ways. The *α*-glucosidases were composed of a series of enzymes, such as maltase, sucrase, lactase, *α*-dextrinase, and *α*-amylase [13, 14]. The *α*-amylase was a class of starch degrading enzymes catalyzing the hydrolysis of internal *α*-1,4-glycosidic bonds in polysaccharides [35]. Starch was hydrolyzed into dextrin, maltose, and a small amount of glucose by the *α*-amylase enzyme [16]. Maltose was composed of two molecules of glucose condensed by *α*-1, 4-glycosidic bonds [36]. Sucrose was made up of a molecule of *α*-glucose and a molecule of *β*-fructose connected by *α*-(1,2)-*β*-glycosidic bonds [37]. The disaccharides of maltose and sucrose were hydrolyzed into monosaccharides by the effect of maltase and sucrase enzymes, respectively [18]. In this study, significant reductions were observed on the postprandial hyperglycemia of TZQ following the ingestion of starch, maltose, and sucrose, which was consistent with the results predicted in the literatures [6, 7, 27–30]. Glucose was directly transported by various transporters embedded on the membranes of enterocytes and absorbed into the blood circulation system. It did not need the hydrolyzing effect of *α*-glucosidase enzymes [14]. The results indicated that TZQ might retard the absorption of glucose by inhibiting the breakage of *α*-(1,2)-*β*-glycosidic bonds in sucrose and the breakage of *α*-1,4-glycosidic bonds in starch and maltose. The polysaccharide of maltodextrin was an incomplete hydrolysis product of starch with a dextrose equivalent value of less than 20%. Maltodextrin was hydrolyzed into *α*-limit dextrin, maltose, and glucose with the hydrolyzing effect of *α*-dextrinase enzymes [17]. Compared with maltodextrin alone, no significant differences were observed on the IAUC and GI values of glucose after the concurrent administration of maltodextrin with 3-, 6-, or 9-tablet TZQ in this study (*P* > 0.05). It indicated that TZQ had no significant depression effect on the breakage of the *α*-1, 6-glycosidic bonds in the *α*-limit dextrin. The results further confirmed that glucose was hydrolyzed from starch via the hydrolysis pathways of *α*-amylase and maltase, rather than the hydrolysis pathways of *α*-amylase and *α*-dextrinase. Therefore, we speculated that TZQ might retard the absorption of glucose by the inhibition of carbohydrate hydrolyzing enzymes of maltase, sucrase, and *α*-amylase in the digestive organs (Figure 7). The postprandial hypoglycemic mechanisms of TZQ might be similar to the inhibitory activities of acarbose against intestinal sucrase and pancreatic *α*-amylase [38] and the inhibitory activities of voglibose against intestinal sucrase and maltase [39]. A further *in vitro* study will be implemented to determine changes in the activity and secondary structure of the *α*-glucosidases inhibited by TZQ. The main limitation of this study is that the impact of TZQ was observed in healthy subjects. These results cannot be generalized to all populations, in particular to those with glucose intolerance, type 2 DM.

## 5. Conclusions

A randomized crossover trial was performed to prove that the TZQ possessed the acute effects on the postprandial glycemia resulting from sucrose, maltose, and starch by inhibiting the maltase, sucrase, and *α*-amylase in the digestive organs. The TZQ is a potential treatment for postprandial hyperglycemia.

## Figures and Tables

**Table 1:** Randomized list of the administration sequences in each subgroup.

	Subgroup A1 (*n* = 2)^*∗*^	Subgroup A2 (*n* = 2)	Subgroup A3 (*n* = 2)	Subgroup A4 (*n* = 2)	Subgroup A5 (*n* = 1)	Subgroup A6 (*n* = 1)	Subgroup A7 (*n* = 1)	Subgroup A8 (*n* = 1)
Period 1	Glucose	Starch + TZQ	Maltodextrin + TZQ	Sucrose + TZQ	Maltose + TZQ	Glucose + TZQ	Glucose	Glucose
Period 2	Glucose	Glucose	Starch + TZQ	Maltodextrin + TZQ	Sucrose + TZQ	Maltose + TZQ	Glucose + TZQ	Glucose
Period 3	Glucose	Glucose	Glucose	Starch + TZQ	Maltodextrin + TZQ	Sucrose + TZQ	Maltose + TZQ	Glucose + TZQ
Period 4	Glucose + TZQ	Glucose	Glucose	Glucose	Starch + TZQ	Maltodextrin + TZQ	Sucrose + TZQ	Maltose + TZQ
Period 5	Maltose + TZQ	Glucose + TZQ	Glucose	Glucose	Glucose	Starch + TZQ	Maltodextrin + TZQ	Sucrose + TZQ
Period 6	Sucrose + TZQ	Maltose + TZQ	Glucose + TZQ	Glucose	Glucose	Glucose	Starch + TZQ	Maltodextrin + TZQ
Period 7	Maltodextrin + TZQ	Sucrose + TZQ	Maltose + TZQ	Glucose + TZQ	Glucose	Glucose	Glucose	Starch + TZQ
Period 8	Starch + TZQ	Maltodextrin + TZQ	Sucrose + TZQ	Maltose + TZQ	Glucose + TZQ	Glucose	Glucose	Glucose

^*∗*^The influences of 3-, 6-, and 9-tablet TZQ on the blood glucose levels were evaluated following the oral administration of the carbohydrates to subjects in the *low-dose group*, *middle-dose group,* and *high-dose group*, respectively.

**Table 2:** Study participant demographic characteristics (mean ± SD).

Variable	Low-dose group	Middle-dose group	High-dose group
Sex (male/female)	6/6	7/5	6/6
Age (years)	25.83 ± 1.34	26.00 ± 2.45	25.50 ± 1.57
Body mass index (kg/m^2^)	21.89 ± 1.62	21.11 ± 1.58	21.53 ± 1.77
Body temperature (°C)	36.26 ± 0.31	36.33 ± 0.21	36.12 ± 0.15
Pulse (times/min)	78.25 ± 8.74	78.17 ± 8.11	75.58 ± 9.94
Breathing (times/min)	18.58 ± 0.67	19.00 ± 0.95	18.00 ± 1.41
Systolic pressure (mmHg)	115.42 ± 15.88	109.58 ± 13.89	101.25 ± 30.54
Diastolic pressure (mmHg)	73.33 ± 8.88	73.75 ± 9.08	72.50 ± 8.92

## Data Availability

The data used to support the findings of this study are available from the corresponding author upon request.

## Abbreviations

ANOVA: Analysis of variance
BMI: Body mass index
DNJ: 1-deoxynojirimycin
GI: Glycemic index
IACU: Incremental areas under the curve
TZQ: Tangzhiqing tablet.
